# Creatine Kinase Surge: Levetiracetam-Induced Rhabdomyolysis

**DOI:** 10.7759/cureus.66715

**Published:** 2024-08-12

**Authors:** Kaitlyn N Romero, Melville C O'Brien, Avni Agrawal, Irtiza Hasan

**Affiliations:** 1 Internal Medicine, University of Florida College of Medicine – Jacksonville, Jacksonville, USA

**Keywords:** creatine kinase, antiepileptic drug, seizure, levetiracetam side effect, rhabdomyolysis

## Abstract

Rhabdomyolysis is the breakdown of muscle cells secondary to both traumatic and non-traumatic causes. The lysing of the muscle cells can lead to the release of cell contents that can lead to acute injury and other life-threatening conditions. Levetiracetam is an anticonvulsant commonly used in generalized and partial tonic-clonic seizures. Well-known side effects include agitation, depression, anxiety, irritability, rash, and somnolence; however, there are an increasing number of case reports that report rhabdomyolysis secondary to antiepileptic use. We present a case of a 27-year-old male with new-onset seizures who was started on levetiracetam therapy and found to have elevated creatine kinase (CK), which decreased only with tapering of the drug. Our case displays the importance of considering levetiracetam as a cause of rhabdomyolysis, supporting this rare side effect of the antiseizure medication. Rhabdomyolysis is a potentially life-threatening condition that can lead to irreversible renal damage if not managed properly.

## Introduction

Rhabdomyolysis is characterized by muscle injury resulting in the release of intracellular contents including myoglobin, potassium, and creatine kinase (CK) [1]. The myoglobin that is released can be nephrotoxic, leading to acute kidney injury (AKI) from the myoglobin that can obstruct and directly injure renal tubules [1,2]. Rhabdomyolysis is diagnosed with CK levels greater than three to five times the upper limit of normal and myoglobinuria on urinalysis [2]. Rhabdomyolysis is typically classified into traumatic and non-traumatic. Non-traumatic causes include the use of statins, fibrates, psychotropic substances, illicit drugs, antibiotics, and certain herbs [1-3]. Patients will typically present with muscle weakness, myalgia, and dark red urine due to myoglobinuria [2,3]. Ultimately, muscle injury occurs with the release of enzymes, electrolytes, and other intracellular components that can be permanently damaging to the kidneys and lead to life-threatening events, such as fatal cardiac arrhythmias [1-3]. 

Levetiracetam is a second-generation antiepileptic agent used to treat generalized and partial seizures as both monotherapy and adjuvant therapy [4]. The drug modulates neurotransmitter release by binding to the synaptic vesicle protein SV2A in the brain and decreasing vesicle release, although the complete mechanism is not fully understood [4]. The most common adverse effects associated with levetiracetam include agitation, depression, anxiety, irritability, rash, and somnolence. Rarely, pancytopenia, Stevens-Johnson syndrome, suicidal ideation, psychosis, and even liver injury have been associated with its use [4,5]. The drug’s metabolites are primarily excreted through the kidneys and require renal-dosing adjustments in the setting of renal impairment [4]. There have been few case reports showing rhabdomyolysis following initiation of levetiracetam that subsided with discontinuation or decreased dosing of the drug; however, it is not a side effect that is well-known to be associated with levetiracetam, and there is currently no warning of the potential link by drug manufacturers [3,6-7]. 

## Case presentation

The case presents a 27-year-old male with a medical history of spinocerebellar ataxia and type 2 diabetes mellitus who initially presented to the emergency room with abdominal pain. The patient was admitted for management of status epilepticus after sustaining multiple general tonic-clonic-type seizures refractory to benzodiazepine withdrawal. The patient did not experience any falls or trauma. Initial labs were significant for metabolic acidosis with a bicarbonate of 17 mmol/L, lactic acidosis of 6.8 mmol/L, elevated beta-hydroxybutyrate, mild hypokalemia, and CK of 508 U/L. The patient was febrile with a temperature of 104.5°F, blood pressure of 125/75 mmHg, tachycardic to 125 beats per minute in normal sinus rhythm, tachypneic to 32 breaths per minute, and an oxygen saturation of 94% on room air. Urinalysis was significant for moderate blood and glucosuria. Urine microscopy did not visualize red blood cells. Initial laboratory results from admission are shown in Table 1.

**Table 1:** Laboratory Results on Hospital Day One

Complete Metabolic Panel	Value	Reference Range
Sodium	140	135 - 145 mmol/L
Potassium	3	3.3 - 4.6 mmol/L
Chloride	107	101 - 110 mmol/L
Carbon Dioxide	17	21 - 29 mmol/L
Urea Nitrogen	30	6 - 22 mg/dL
Creatinine	0.53	0.51 - 0.96 mg/dL
Blood Urea Nitrogen (BUN)/Creatinine Ratio	29.4	6.0- 22.0 calc
Glucose	373	71 - 99 mg/dL
Calcium	9.2	8.6 - 10.0 md/dL
Total Protein	7.2	6.5 - 8.3 g/dL
Albumin	3.8	3.8 - 4.9 g/dL
Total Bilirubin	0.4	0.2 - 1.0 mg/dL
Alkaline Phosphatase	95	35 - 104 IU/L
Aspartate Transaminase (AST)	24	14 - 33 IU/L
Alanine Transaminase (ALT)	21	10 - 42 IU/L
Anion Gap	16	4 - 16 mmol/L
Estimated Glomerular Filtration Rate (eGFR) (EGFR)	102	≥ 60 mL/min/1.73m2
Creatine Kinase (CK)	508	22 - 195 U/L
Lactic Acid	6.7	0.7 - 2.0 mmol/L
Hemoglobin A1C	6.7	4.8 - 5.9 %
Complete Blood Count and Differential		
White Blood Cell (WBC)	5.13	4.0 - 10.0 x10^3^/µL
Red Blood Cell (RBC)	5.67	4.0 - 5/2 x10^6^/µL
Hemoglobin	14.8	12.0 - 16.0 g/dL
Hematocrit	46.7	35.0 - 45.0 %
Mean Corpuscular Volume (MCV)	82.4	78.0 - 100.0 fl
Mean Corpuscular Hemoglobin (MCH)	26.1	26.0 - 34.0 pg
Mean corpuscular hemoglobin concentration (MCHC)	31.7	31.0 - 36.0 g/dL
Red Cell Distribution Width (RDW)	13.8	11.0 - 14.6%
Platelet Count	176	150 - 450x10^3^/µL
Mean Platelet Volume (MPV)	9.5	9.5 - 12.2 fl
Neutrophil %	77.8	34 - 73%
Bands %	17.9	0 - 10%
Lymphs %	9.9	25 - 45%
Monocytes %	11.7	2 - 6%
Metamyelocytes %	0	≤ 0%
Myelocytes %	0	≤ 0%
Promyelocytes %	0	≤ 0%
Neutrophil Absolute	7.9	1.4 - 7.5x10^3^/µL
Lymphocyte Absolute	0.51	0.7 - 3.1x10^3^/µL
Monocytes Absolute	0	0.1 - 0.9x10^3^/µL
Immature Granulocytes Absolute	0	≤ 0.0x10^3^/µL

The patient was admitted to the intensive care unit due to status epilepticus and further monitoring for seizures, loaded with levetiracetam 1,500 mg, and started on 1,000 mg twice daily for maintenance dosing. The patient did not require intubation, and further seizures were electrographically detected, characterized by staring episodes with no further convulsions. The etiology of the seizure was attributed to possible benzodiazepine withdrawal as the patient was on clonazepam in the outpatient setting.

On day two of the patient’s hospitalization, the CK trended up to 1,100 U/L and continued to increase rapidly and peaked at 40,673 U/L on day four. Intravenous fluid replacement began on day three, with normal saline at 150 ml/hr. Nephrology was consulted on day four due to the increasing CK, refractory to fluid hydration of greater than eight L of lactated ringers solution over two days. Creatinine was 1.0 mg/dL, elevated from the baseline renal function of the patient of 0.53 mg/dL on admission, significant for an AKI. Nephrology recommended continuing IV hydration and trend CK daily as further workup was being conducted for the etiology of the AKI. Despite an initial decrease of CK to 32,000 U/L on days five to seven, CK increased on day eight to 37,000 U/L. Nephrology recommended decreasing the dose of levetiracetam due to a few cases of rhabdomyolysis with the initiation of levetiracetam. The dose was changed from 1,000 mg twice a day (BID) to 500 mg BID. On day nine, CK significantly decreased to 23,300 U/L and continued to downtrend to under 3,000 U/L on day 12 and eventually normalized. Throughout the entire admission, the patient averaged 1.5-2 liters of urine output daily, and the AKI resolved. 

The remainder of the hospital course included an electroencephalogram that revealed no epileptiform activity but background slowing suggestive of a mild to moderate encephalopathy. A lumbar puncture was conducted. Meningitis was ruled out with negative cerebrospinal fluid studies for herpes simplex virus, varicella-zoster virus, and negative gram stain. There was an initial concern for an inflammatory myopathy, but the rhabdomyolysis resolved with the reduction in the dose of levetiracetam; thus, further rheumatological and autoimmune work-up was not performed.

At the two-month follow-up neurology appointment, the patient and family denied further seizures. A basic metabolic panel demonstrated renal function was stable (creatinine 0.6 mg/dL). The patient was recommended to continue on levetiracetam 500 mg BID.

## Discussion

Although levetiracetam is not known to be associated with rhabdomyolysis, our case adds to the case reports over the last few years that present similar clinical courses. There have not been any large studies showing a strong association between levetiracetam and rhabdomyolysis, but there is a growing number of case reports and case series further supporting that a rare side effect of levetiracetam is muscle injury [3, 6-7]. The exact mechanism is currently unknown. A retrospective case analysis used the United States FDA Adverse Event Reporting System database (FAERS) and found that there were 1,100 reported rhabdomyolysis cases associated with newer generation antiseizure medications as well as case reports, the majority being reported with the use of levetiracetam. However, these are self-reported events that can be confounded with other causes of rhabdomyolysis. In the same study of 26 case reports, 11 were in the setting of levetiracetam use, with other antiseizure medications such as gabapentin, pregabalin, lamotrigine, and lacosamide [3]. 

**Figure 1:**
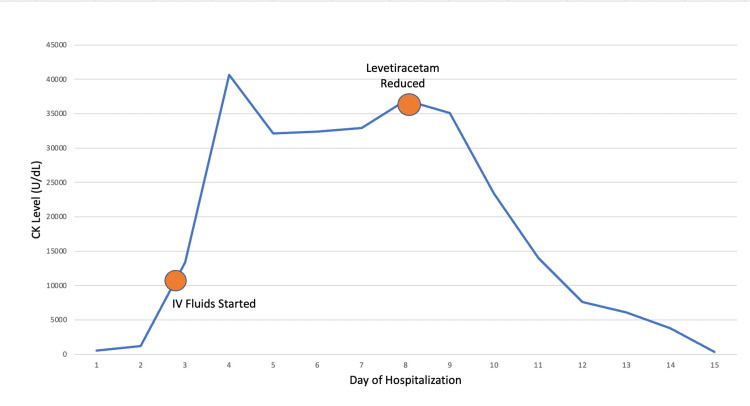
Rhabdomyolysis with Rapid Resolution Following Decrease in Levetiracetam CK: Creatine Kinase; IV: Intravenous; Orange Circles Represent the Time of the Event

Autoimmune and rheumatologic workups were not done in this patient as the rhabdomyolysis resolved once levetiracetam was removed, but other causes of elevated CK, particularly in the setting of no kidney injury, should be considered. Elevated CK levels can be seen in several different situations, such as crush injuries, exercise, immobility, seizures, inflammatory myopathies, drug use, medications, metabolic and mitochondrial disorders, and muscular dystrophies [1, 2, 8]. The patient in our case was admitted due to seizures and was found to have a mildly elevated CK that continued to increase despite the cessation of convulsions in the setting of seizures and IV fluid initiation. The CK level was inappropriately elevated for multiple days, as seen in Figure 1. The patient was primarily bed-bound and immobile but had a history of normal CK with the same functional status in multiple previous admissions per chart review.

Levetiracetam was introduced on hospital day one in the neurological ICU at 1,000 mg twice daily given the patient's presentation in status epilepticus. The initial etiology was thought to be benzodiazepine withdrawal, as the patient's primary care physician was tapering down his frequency of benzodiazepine use for anxiety. Day two to day four displayed a significant increase in CK that decreased with the initiation of crystalloid fluids from 40,000 U/dL to 32,000 U/dL. However, an increase in CK occurred from day four through day eight, while other workups remained unremarkable. Levetiracetam was reduced on day eight, with a rapid reduction in CK through his hospitalization, and normalized in the outpatient setting. The patient had no further convulsions or muscular trauma, and no other medications were administered known to cause rhabdomyolysis. An autoimmune etiology was not suspected given the patient's acute presentation and lack of other associated symptoms of myositis. Thorough medication review and broad differential diagnosis are important when common etiologies are not found to be a clear cause of elevated CK, as severe renal damage can occur and lead to a need for hemodialysis [1-2, 8]. The patient developed an AKI on day two but was not found to have manifestations of renal failure secondary to rhabdomyolysis such as hyperkalemia, uremia, and acidemia that can lead to the need for hemodialysis. 

Further studies should focus on a dose-dependent component of levetiracetam and its potential cause of rhabdomyolysis. In our case, the patient was loaded with a high dose of levetiracetam and then placed on a twice-daily dosing. A maximum of 4,000 mg of levetiracetam is recommended and split over two doses [5]. Patients are on varying doses between 500 and 1,000 mg daily and receive higher doses depending on being used as an abortive or preventive therapy [6,7]. Studies should be done to assess the association of rhabdomyolysis in patients receiving loading versus maintenance doses.

## Conclusions

There is a rising number of case reports that associate antiepileptic agents, specifically levetiracetam, with rhabdomyolysis. In our case, we were able to exclude both traumatic and atraumatic causes of rhabdomyolysis that initially confounded the clinical picture. Levetiracetam was found to be the cause of rhabdomyolysis in this patient after decreasing its dose and seeing a resolution of increasing CK. Rhabdomyolysis is related to several causes, which emphasizes the importance of conducting an extensive workup and keeping a broad differential diagnosis to avoid the harmful effects of rhabdomyolysis. 
